# Morphological Characteristics of Normal Foveal Avascular Zone by Optical Coherence Tomography Angiography

**DOI:** 10.1155/2020/8281459

**Published:** 2020-08-19

**Authors:** Zeiad Eldaly, Wael Soliman, Mohamed Sharaf, Ali Natag Reyad

**Affiliations:** ^1^Department of Ophthalmology, Assiut University, Assiut, Egypt; ^2^Alforsan Center, Assiut, Egypt

## Abstract

**Purpose:**

To provide quantitative measurements for the foveal avascular zone (FAZ) and to describe its morphological characteristics by optical coherence tomography angiography (OCT-A).

**Design:**

Cross-sectional observational case series.

**Methods:**

Healthy volunteers were recruited and evaluated using Triton-DRI SS-OCT Angiography. A 4.5 × 4.5 mm square was evaluated by OCT-A center around the fovea. Superficial and deep capillary plexus were identified, and different quantitative measurements were conducted along with categorization of the FAZ pattern.

**Results:**

Eighty-two eyes (41 volunteers) were evaluated. Mean age was 30.59 ± 7.6 years (23–42 years). Mean subcentral retinal thickness was 200.1 ± 5.66 um (192–210 um). The number of terminal vessels was variable (range, 8–11). Mean maximum terminal vessel-to-vessel intervening distance was 527.8 ± 60.3 um (471–674 um). Mean minimum terminal vessel-to-vessel intervening distance was 296.7 ± 45.8 um (233–373 um). Mean maximum horizontal diameter of FAZ (superficial) was 716.17 ± 124.09 um, while mean maximum vertical diameter of FAZ (superficial) was 667.76 ± 131.28 um. Mean maximum horizontal diameter of FAZ (deep) was 823.19 ± 144.92 um, while mean maximum vertical diameter of FAZ (deep) was 794.03 ± 150.28 um. OCT-A detected different FAZ patterns; horizontally oval configuration in 32 eyes (39%), rounded configuration in 24 eyes (29.3%), pentagon configuration in 14 eyes (17.1%), and vertically oval and nonspecific configuration in 6 eyes each (7.3%).

**Conclusion:**

OCT-A could efficiently provide both quantitative and qualitative assessment of normal FAZ; such characterizations were difficult by standard FAZ assessment procedures like FFA.

## 1. Introduction

Macular perfusion status is altered by different retinal vascular diseases, most importantly diabetic retinopathy and retinal vascular occlusion [1, 2]. Macular ischemia is often diagnosed by disruption and irregularities of foveal avascular zone (FAZ) which was thoroughly evaluation by fundus fluorescein angiography (FFA) [3, 4]. Quantitative evaluation of the FAZ was conducted by utilizing FFA; however, FFA could only highlight the superficial FAZ not deeper vascular layers [5]. In addition, associated media opacity, lack of patient cooperation, presence of coexisting macular odema, and failure to acquire early FFA frames will hinder proper evaluation FAZ. In addition, renal failure and dye allergy will also contraindicate the use of FFA in the evaluation of macular perfusion [6].

The advent of optical coherence tomography angiography (OCT-A) provided retina physicians with noninvasive, dyeless, and in-depth evaluation of FAZ. [7] Furthermore, OCT-A could assess not only the superficial capillary plexus and FAZ but also the deep capillary plexus and its FAZ [8, 9]. Evaluation of deep capillary plexus and its FAZ could not be achieved by FFA and made possible only by the technology of OCT-A [10]. Manual measurement of superficial and deep FAZ is available in several devices, and other devices offered automatic measurements of FAZ [11].

Several studies had described the morphological characteristics of the FAZ in healthy subjects including FAZ area with contradicting correlation with ocular and demographic data. [12–14] Several studies had reported detailed measurements of the FAZ as FAZ diameters. [10, 12] The aim of the study was to provide a detailed quantitative evaluation of both superficial and deep FAZ, to categorize different FAZ patterns and to clarify their relation to different ocular and demographic data characteristics.

## 2. Methods

### 2.1. Study Design

A cross-sectional observational case series study was performed from November 2016 till April 2018. The study was done in accordance with the Declaration of Helsinki and after obtaining the approval of Institutional Review Board of the Faculty of Medicine, Assiut University. Discussion of the procedure details with the volunteers was done, and informed consent was obtained.

### 2.2. Participants

Healthy volunteers were recruited after complete ophthalmic and systemic evaluation. All participants underwent best-corrected distance visual acuity (d-BCVA) by Snellen's chart, slit lamp assessment of the anterior segment, dilated fundus examination, and intraocular pressure (IOP) measurement by calibrated Goldmann Applanation Tonometer (GAT). Participants with normal ophthalmic assessment and absent systemic illness were included. Any participant with past history of ocular disease, systemic illness, previous ocular or refractive surgery, myopia more than 6 diopters, or participant age at recruitment date less than 18 years were excluded. Any participant with poor fixation interfering with OCT-A acquisition was excluded according to operator discern.

### 2.3. OCT Acquisition and Scan Properties

Included volunteers were evaluated by Swept Source OCT (Triton Deep Range Imaging (DRI) SS-OCT), with long wavelength scanning light (1050 nm). During each imaging session, 2 sets of OCTA scans were obtained for a 4.5 × 4.5 mm square centered upon the fovea. It operated with an optimized long wavelength of examination light (1050 nm). It also performs 100,000 A-scans per second with a lateral resolution of 20 *μ*m and in-depth resolution of 2.6 *μ*m. Automatic correction of minor artifacts was carried out by the OCT-A software. OCT-A images with gross motion artifacts, blink artifact, segmentation artifacts, or projection artifact as described by Spaide et al. [15] were repeated until satisfactory scan quality was guaranteed. The study depended on automatic segmentation of superficial and deep capillary plexus performed the device. The superficial capillary plexus had its upper boundary located 3 *μ*m below the internal limiting membrane (ILM) and lower boundary located 15 *μ*m below the inner plexiform layer (IPL). Meanwhile, deep capillary plexus had its upper boundary 15 um below IPL and its lower boundary located 70 um below IPL. Standard OCT and OCT-A were done by a single experienced operator (Z. E.), while measurement and analysis were done by 2 independent physicians (M. S. and W. S.).

**Figure 1:**
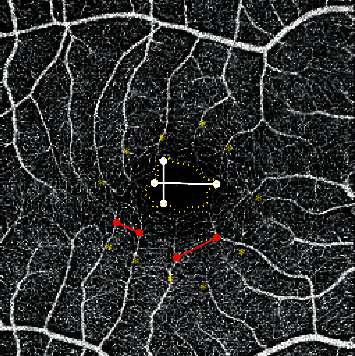
FAZ measurements: FAZ area (dashed yellow line), minimum and maximum intervening distances (red lines), maximum vertical and horizontal diameters (white lines), and number of terminal vessels (asterisks).

Subcentral retinal thickness was measured from the ILM to the outer border of RPE-Bruch membrane complex, while subcentral choroidal thickness was measured from the outer border of RPE-Bruch membrane complex to the chorioscleral interface (CSI). Evaluation parameters of OCT-A images (Figure 1) included the number of terminal vessels contributing in the formation of FAZ in the superficial retinal plexus, maximum and minimum vessel-to-vessel intervening distance, largest horizontal and vertical diameters of FAZ, area of FAZ in superficial and deep plexus, and ratio between FAZ area measured in superficial and deep plexus. The pattern of FAZ by OCT-A was also classified into rounded, vertically oval, horizontally oval, pentagon, and nonspecified. After measurement of different FAZ parameters, the average of the readings obtained by the 2 independent physicians was calculated provided that the difference between two readings does not exceed 25% of any of them. If the difference exceeds such limit, remeasurement will be done and mean will be calculated directly.

### 2.4. Statistical Analysis

Statistical analysis was carried out with SPSS, Version 20 (SPSS Inc, Illinois, USA). The Mann-Whitney test was utilized to compare the means among groups, while the Pearson correlation coefficient was used to assess correlation between groups. A *p* value less than 0.05 was considered statistically significant.

## 3. Results

### 3.1. Demographic Characteristics and Baseline Evaluation

Eighty-two eyes (41 volunteers) were evaluated. There were 23 male (56.1%) and 18 (43.9%) female. Mean age was 30.59 ± 7.6 years (23–42 years). No abnormality was found on standard OCT evaluation. Mean subcentral retinal thickness was 206.85 ± 7.33 um (95% CI: 204.29–209.41 um). Mean subcentral choroidal thickness was 352.08 ± 62.02 um (95% CI: 330.44–373.72 um).

### 3.2. FAZ Parameters

Analysis of the images of both superficial and deep retinal plexus revealed that the configuration of both layers is different. In superficial retinal plexus, the border of the FAZ is well-delineated, and vascular network distribution is coarse and related to retinal blood vessel distribution. Meanwhile, the border of FAZ in deep retinal plexus is indistinct with fine and compact distribution of its vascular network.

**Figure 2:**
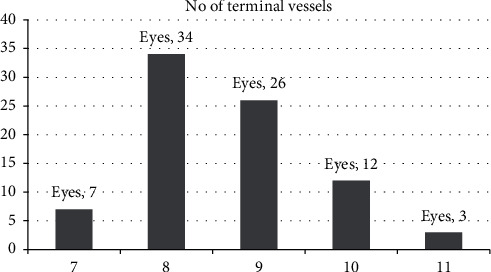
Distribution of the number of terminal vessels (superficial plexus) by OCT-A.

The number of terminal vessels was variable, ranging from 7 to 11 terminal vessels (Figure 2). Mean maximum vessel-to-vessel intervening distance was 554.58 ± 91.26 um (95% CI: 522.74–586.43 um). Mean minimum vessel-to-vessel intervening distance was 267.64 ± 62.83 um (95% CI: 245.72–289.57 um). Mean maximum horizontal diameter of FAZ (superficial) was 716.17 ± 124.09 um (95% CI: 672.87–759.47 um), while mean maximum vertical diameter of FAZ (superficial) was 667.76 ± 131.28 um (95% CI: 621.95–713.57 um). There was a statistically significant difference between the horizontal and vertical diameter of FAZ (superficial) (*p* value 0.012). Mean maximum horizontal diameter of FAZ (deep) was 823.19 ± 144.92 um (95% CI: 702.27–1019.33 um), while mean maximum vertical diameter of FAZ (deep) was 794.03 ± 150.28 um (95% CI: 649.83–993.34 um). There was no statistically significant difference between the horizontal and vertical diameter of FAZ (deep) (*p* value 0.712).

Mean area of FAZ in superficial plexus was 386.41 ± 108.48  um^2^ (95% CI: 348.56–424.26 um^2^) while mean area of FAZ in deep plexus was 463.52 ± 100.94 um^2^ (95% CI: 402.30–547.75 um^2^). There was no statistically significant difference between FAZ area at superficial and deep retinal plexus (*p* value 0.872). The mean ratio between FAZ area measured in superficial and deep plexus was 0.833 ± 0.114 (range from 0.722–0.942, 95% CI: 0.784–1.023).

**Figure 3:**
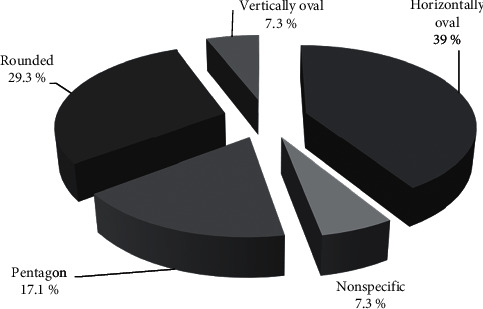
Distribution of the FAZ pattern (superficial plexus) by OCT-A.

**Figure 4:**
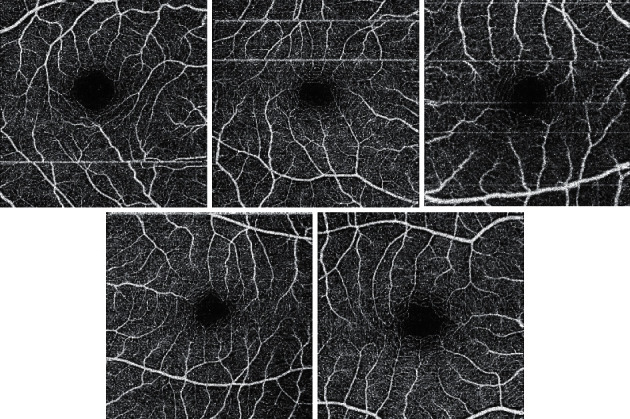
FAZ pattern (superficial plexus) by OCT-A. Top right: vertically oval; top middle: horizontally oval; top left: rounded; bottom right: pentagon; bottom left: nonspecified.

The pattern of FAZ by OCT-A was variable, the horizontally oval configuration was found in 32 eyes (39%), the rounded configuration was found in 24 eyes (29.3%), the pentagon configuration was found in 14 eyes (17.1%), the vertically oval configuration was found in 6 eyes (7.3%), and nonspecified configuration was found in 6 eyes (7.3%). Distribution of FAZ pattern is summarized in Figures 3 and 4.

### 3.3. Correlation of FAZ Measurements with Retinal and Choroidal Thicknesses

Superficial and deep retinal plexus areas directly, strongly, and significantly correlate with each other (Pearson correlation coefficient: 0.929; *p* value 0.0001). Neither superficial nor deep FAZ area correlated significantly with age (*p* values 0.648 and 0.572 respectively). In addition, both horizontal and vertical FAZ diameters (measured at the level of superficial retinal plexus) were not correlating significantly with age. FAZ areas (superficial and deep retinal plexus) and superficial/deep FAZ area ratio are inversely correlating with subcentral retinal thickness (*p* values 0.004, 0.033, and 0.045, respectively) (Table 1). No significant correlation was found between superficial, deep FAZ area, and superficial/deep FAZ area ratio with subcentral choroidal thickness (Table 2). Maximum horizontal FAZ diameter is directly correlating with FAZ areas (superficial and deep retinal plexus) and superficial/deep FAZ area ratio (Table 3). Maximum vertical FAZ diameter is directly correlating with FAZ areas (superficial and deep retinal plexus) (Table 4).

### 3.4. Gender Difference in FAZ Measurements

Females had significantly large superficial and deep FAZ areas. Though insignificant, females had larger vertical and horizontal diameters in both superficial and deep FAZ except horizontal diameter in deep FAZ.  Table 5 summaries the gender difference in FAZ measurements.

## 4. Discussion

The intact microvascular structure of the FAZ is critical for providing a satisfactory visual function. Visual impairment resulting from ischaemic maculopathy secondary to diabetic retinopathy or retinal venous occlusions is clearly associated with disruption of the FAZ architecture. [1, 2] FFA remained for decades the golden standard for the assessment of the FAZ, and it provided generous data regarding the integrity of FAZ. Other imaging modalities had been used for FAZ visualization. [16] The spectrum of the disruption involving the FAZ includes enlargement of the FAZ area or diameter, disruption of terminal vessels, and widening of the space between terminal vessels. [1, 2].

OCT angiography provided a dyeless noninvasive comprehensive evaluation of the retinal and choroidal circulation. It provided a clear visualization of superficial and deep capillary plexuses along with evaluation of the avascular outer retina and the choriocapillaries layer. [17, 18] Park and colleagues identified a middle capillary plexus by OCT angiography. [19] On the other hand, FFA could only contribute to the evaluation of the superficial capillary plexus and FAZ. This could be attributed to light scattering by different retinal layers. Wu and associates evaluated superficial FAZ area by FFA (0.43 mm^2^) and superficial FAZ maximum diameters (horizontal 0.73 mm and vertical 0.70 mm). [5] John and co-workers showed different FAZ area measurements using contrast-adjusted and nonadjusted methods of measurements by FFA. In the contrast-adjusted method, FAZ area was significantly smaller than that of the nonadjusted method (0.275 mm^2^ versus 0.624 mm^2^, respectively) [20].

In the current study, detailed measurements of both superficial and deep FAZ were rendered. OCT angiography revealed that 42% of eyes had 8 terminal vessels and 32% of eyes had 9 terminal vessels in superficial FAZ. The distance between terminal vessels is one of the early findings in macular ischeamia. Maximum intervening distance between terminal vessels in superficial FAZ was 554 um, while minimal distance was 245 um.

Several studies had provided superficial and deep FAZ areas. In the current study, superficial FAZ area was 386 um^2^ and 463 um^2^ in deep FAZ. Tan and associates reported that superficial and deep FAZ areas were 0.24 mm^2^ and 0.38 mm^2^, respectively. [13] Shahlaee and contributors also highlighted that superficial FAZ area was 0.27 mm^2^ and deep FAZ area was 0.34 mm^2^. [21] Ghassemi and co-workers provided comparable superficial FAZ area and deep FAZ area to previous reports (0.27 mm^2^ and 0.35 mm^2^, respectively). [22] Our study reported greater FAZ area in both superficial and deep capillary plexus; however, we concur with those reports that deep FAZ is wider than the superficial one. Several studies provided superficial area only by OCT angiography with comparable measurements as researchers find some difficulties in clearly delineating the boundaries of the deep FAZ with great interobserver variability in deep FAZ measurement in contrast to reproducible superficial FAZ outline. [21] Superficial FAZ area was 0.47 mm^2^ found by Di et al., 0.35 mm^2^ by Yu et al., and 0.36 mm^2^ by Wang et al. [10, 14, 23].

In our study, the mean vertical diameter of superficial FAZ was 667 um with a significantly larger diameter in deep FAZ 794 um. Similarly, horizontal diameter in deep FAZ was significantly larger than the superficial one (823 um versus 716 um, respectively). Di and colleagues reported superficial FAZ diameters only, the vertical diameter was 0.33 mm, and horizontal diameter was 0.35 mm. [10] The vertical and horizontal diameters of superficial FAZ were 0.56 mm and 0.59 mm, respectively, and in deep FAZ were 0.63 mm and 0.69 mm, respectively, as reported by Shahlaee et al. [21] Hussain and collaborators measured only vertical and horizontal diameters of superficial and deep FAZ and not FAZ area. In the superficial FAZ, the vertical diameter was 660 um and horizontal diameter was 661 um, while in deep FAZ, vertical diameter was 818 um and horizontal diameter was 1011 um. [24] The difference of FAZ measurements among investigators could be attributed to variable selection criteria, ethnic and gender differences, various OCT-A devices, and acquisition protocols. [25] Only Tan and colleagues shed some light upon FAZ pattern with no specific categorization. [13] In the current study, we highlighted the patterns of superficial FAZ. Horizontally oval superficial FAZ was the most common pattern (39% of eyes). About one third the eyes had rounded pattern, while pentagon shape was found in 17%.

No correlation between FAZ parameters and age in the current study was found. Tan et al. and Wang et al. also highlighted no significant correlation with age. [13, 23] However, Ghassemi and colleagues reported a significant correlation between superficial and deep FAZ areas and age [22], and FAZ area was found to increase annually by 1.48% as reported by Yu et al. due to senescence of the retinal vascular network. [14] In our study, females had larger horizontal and vertical diameters in both superficial and deep FAZ. Furthermore, FAZ area was greater in females than in males as reported by other different studies. [13, 14, 22] However, Samara et al. reported no gender difference in FAZ area. [12] No significant correlation was found between FAZ area and choroidal thickness. On the other hand, a significant inverse correlation of FAZ area was found with central retinal thickness. Limitations of the current study are small sample size for a normative data and variability of refractive error.

## 5. Conclusion

Understanding the normal morphological features of FAZ will provide us a better and wider understanding of retinal vascular disorders affecting the macula. Detailed quantitative evaluation of FAZ parameters could provide an insight of earlier signs of macular ischeamia than traditional qualitative FAZ enlargement or irregularities.

## Figures and Tables

**Table 1:** Correlation of subcentral retinal thickness with superficial FAZ area, deep FAZ area, and superficial/deep FAZ area ratio.

	Superficial FAZ area	Deep FAZ area	Superficial/deep FAZ area ratio
Pearson correlation coefficient	−0.479	−0.367	−0.345
*p* value	0.004^*∗*^	0.033^*∗*^	0.045^*∗*^

^*∗*^Significant difference (*p* value < 0.05).

**Table 2:** Correlation of subcentral choroidal thickness with superficial FAZ area, deep FAZ area, and superficial/deep FAZ area ratio.

	Superficial FAZ area	Deep FAZ area	Superficial/deep FAZ area ratio
Pearson correlation coefficient	−0.034	−0.115	0.157
*p* value	0.847	0.518	0.376

^*∗*^Significant difference (*p* value < 0.05).

**Table 3:** Correlation of maximum horizontal FAZ diameter with superficial FAZ area, deep FAZ area, and superficial/deep FAZ area ratio.

	Superficial FAZ area	Deep FAZ area	Superficial/deep FAZ area ratio
Pearson correlation coefficient	0.876	0.757	0.451
*p* value	0.0001^*∗*^	0.0001^*∗*^	0.007^*∗*^

^*∗*^Significant difference (*p* value < 0.05).

**Table 4:** Correlation of maximum vertical FAZ diameter with superficial FAZ area, deep FAZ area, and superficial/deep FAZ area ratio.

	Superficial FAZ area	Deep FAZ area	Superficial/deep FAZ area ratio
Pearson correlation coefficient	0.876	0.842	0.215
*p* value	0.0001^*∗*^	0.0001^*∗*^	0.221

^*∗*^Significant difference (*p* value < 0.05).

**Table 5:** Difference of FAZ measurements according to gender.

	Male	Female	*p* value
Max. horizontal diameter (superficial)	693.26	739.08	0.321
Max. vertical diameter (superficial)	592.11	743.41	0.035^*∗*^

Max. horizontal diameter (deep)	856.83	789.55	0.124
Max. vertical diameter (deep)	778.52	809.54	0.053
Mean superficial FAZ area	360.52	412.3	0.048^*∗*^

Mean deep FAZ area	446.89	480.15	0.034^*∗*^
Superficial/deep FAZ area ratio	0.807	0.859	0.424

^*∗*^Significant difference (*p* value < 0.05).

## Data Availability

The data used to support this study are available upon request.
